# Vertebral Artery Dissection Following High Velocity Low Amplitude Cervical Manipulation: A Case Report

**DOI:** 10.7759/cureus.87689

**Published:** 2025-07-10

**Authors:** Kevin Szafran, YuanDian Zheng, Forrest Butensky

**Affiliations:** 1 Physical Medicine and Rehabilitation, American University of the Caribbean School of Medicine, Florida, USA; 2 Family Medicine, Nassau University Medical Center, New York, USA; 3 Physical Medicine and Rehabilitation, Nassau University Medical Center, East Meadow, USA

**Keywords:** cervical spine manipulation, expressive aphasia, middle cerebral artery aneurysm, traumatic vertebral artery dissection, vertebrobasilar ischemia

## Abstract

Vertebral artery dissection (VAD) is a rare but potentially serious cause of posterior circulation stroke, occasionally associated with high-velocity, low-amplitude cervical spinal manipulation therapy (CSMT). We present the case of a 70-year-old female who developed acute expressive aphasia following chiropractic neck manipulation. Imaging revealed a proximal right vertebral artery occlusion with findings suggestive of dissection in the setting of vascular hypoplasia. The patient was managed conservatively with blood pressure control, antithrombotic therapy, and rehabilitation, ultimately returning to near-baseline function. This case highlights the importance of recognizing VAD as a potential complication of cervical manipulation and underscores the value of early diagnosis, thorough vascular imaging, and multidisciplinary management.

## Introduction

Vertebral artery dissection (VAD) is a rare but clinically important cause of posterior circulation ischemic stroke. It may occur spontaneously or be triggered by trauma, connective tissue disorders (e.g., Ehlers-Danlos, Marfan syndrome), cervical spine instability, or intrinsic vascular abnormalities [1]. Cervical spinal manipulation therapy (CSMT), particularly high-velocity, low-amplitude (HVLA) techniques performed by chiropractors and manual therapists, is a debated potential risk factor [2].

While several studies suggest a temporal association between CSMT and VAD, establishing causality remains challenging due to confounding factors such as underlying vascular fragility, minor trauma from routine activities, or protopathic bias, where symptoms of an evolving dissection (e.g., neck pain) prompt individuals to seek manipulation [3]. Nevertheless, mechanical strain during cervical manipulation-especially with excessive rotation or hyperextension, can compromise the vertebral arteries, which traverse the transverse foramina of C6-C1 and loop around the atlas, making them anatomically susceptible [4].

Injury to the vessel wall may result in intimal tearing, thrombus formation, and embolic stroke. Presenting symptoms range from neck pain and occipital headache to vertigo, ataxia, dysarthria, and, in severe cases, locked-in syndrome or death due to basilar artery involvement [5].

Prompt diagnosis is critical. CT angiography (CTA) is the preferred initial imaging modality, while MRI/MRA with fat-saturated T1-weighted sequences can visualize intramural hematoma. Digital subtraction angiography (DSA) remains the gold standard in select cases [6]. Treatment involves antithrombotic therapy, blood pressure control, and supportive care; endovascular intervention may be considered in refractory cases.

We report a case of VAD following chiropractic cervical manipulation in a previously healthy adult who presented with isolated expressive aphasia and no cervical pain atypical presentation that may delay recognition. Unlike most documented cases, our patient demonstrated significant neurological recovery through conservative management and comprehensive rehabilitation. This report contributes to the growing literature on post-CSMT VAD and emphasizes the importance of early neuroimaging, even in the absence of classic posterior circulation symptoms. It also highlights the need for continued clinical vigilance and a nuanced understanding of this controversial and potentially preventable etiology of stroke.

## Case presentation

Patient information

A 70-year-old right-handed female with a past medical history of well-controlled hypertension and hyperlipidemia presented to the emergency department (ED) after developing sudden expressive aphasia during a chiropractic neck adjustment. She had no known personal or family history of cerebrovascular disease, connective tissue disorders, or migraines. She was a lifelong non-smoker, consumed alcohol occasionally, and was retired and living independently. There were no known drug allergies or relevant genetic conditions.

History of present illness

The patient was undergoing a high-velocity, low-amplitude (HVLA) cervical spinal manipulation for subacute neck discomfort that had been present for several months. This was her first session with this chiropractor, and no formal screening or neurologic assessment was performed before the procedure.

Immediately following the cervical manipulation, the chiropractor noted a sudden change in the patient’s speech. The patient became aware of difficulty forming words but remained fully conscious and oriented. Emergency medical services were activated, and she was transported to the ED approximately 30 minutes after symptom onset. Prior to this event, she was fully independent in all activities of daily living, ambulatory without assistance, and had no prior neurologic deficits.

Clinical examination

On arrival, the patient was alert and oriented to person, place, and time. She exhibited isolated expressive aphasia, characterized by impaired fluency and word-finding difficulty, but preserved comprehension. Motor strength was 5/5 in all extremities, and sensation was intact. Deep tendon reflexes were 1+ bilaterally. Upper motor neuron signs (Babinski, clonus, Hoffman's) were absent. Cranial nerve examination was normal. No dysarthria, facial droop, vertigo, diplopia, ataxia, or dysphagia were noted. The remainder of the neurologic and systemic examinations was unremarkable.

Diagnostic workup

**Figure 1:**
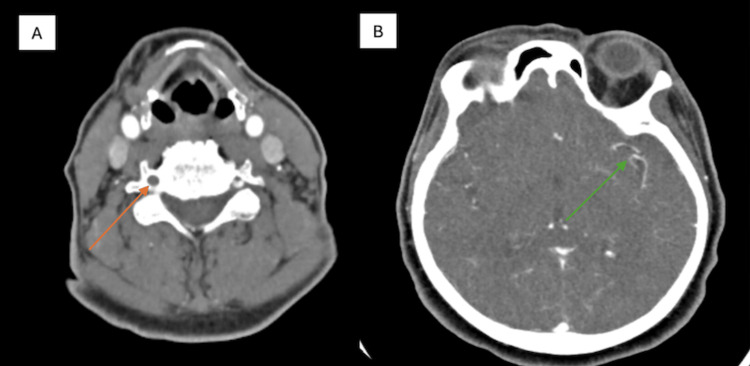
Axial CT angiography of the head and neck. (A) Right vertebral artery dissection at the level of C3 (orange arrow), with a patent left vertebral artery and preserved blood flow. (B) 3.5 mm saccular aneurysm at the bifurcation of the left middle cerebral artery (MCA) (green arrow). CT = computed tomography; C3 = third cervical vertebra; MCA = middle cerebral artery.

CTA of the head and neck revealed complete occlusion of the proximal right vertebral artery at the C3 level (Figure 1A). These findings raised suspicion for acute dissection versus chronic atherosclerotic occlusion. An incidental 3.5 mm saccular aneurysm was identified at the bifurcation of the left middle cerebral artery (MCA) (Figure 1B).

Management and outcomes

Given the low NIH Stroke Scale score, absence of disabling deficits, and anatomical location of the occlusion, conservative management was pursued with no indication for thrombolytic therapy (tPA). Antihypertensive medications were resumed, and the patient was started on daily aspirin (81 mg), with a target systolic blood pressure of less than 140 mmHg. Endovascular intervention was deferred due to the patient’s clinical stability, the non-dominant nature of the affected vertebral artery, and the presence of a patent left vertebral artery with adequate basilar perfusion.

Speech therapy evaluation revealed mild to moderate expressive aphasia without evidence of dysphagia, and speech-language rehabilitation was initiated. Physical and occupational therapy focused on improving endurance, strength, and functional mobility.

Over the course of one week, the patient demonstrated steady clinical improvement. By the time of discharge, she was ambulatory without assistance, independent in activities of daily living, and exhibited only mild residual aphasia with subjective symptom improvement. She was discharged home with outpatient speech therapy and neurology follow-up, along with recommendations for serial imaging to monitor the middle cerebral artery aneurysm.

## Discussion

This case highlights the potential vascular complications associated with high-velocity, low-amplitude (HVLA) cervical spinal manipulation therapy (CSMT), particularly vertebral artery dissection (VAD). Although rare, VAD is a critical diagnosis due to its association with posterior circulation strokes, which may present with subtle neurological deficits or lead to devastating outcomes. In this patient, the sudden onset of expressive aphasia following cervical manipulation, in the absence of other focal deficits, prompted a thorough vascular workup that revealed a proximal right vertebral artery occlusion with imaging features suggestive of dissection or thrombosis in a hypoplastic vessel.

While a definitive cause-and-effect link between CSMT and VAD remains controversial, a growing body of literature suggests a close chronological association [7, 8]. The vertebral arteries, particularly as they navigate through the transverse foramina of the upper cervical spine and curve around the atlas (C1), are susceptible to mechanical strain from neck rotation, extension, or lateral flexion movements commonly induced during chiropractic manipulation. Anatomical anomalies, such as congenital hypoplasia, as seen in this case, may further predispose individuals to vascular injury.

Given the acute onset of isolated expressive aphasia immediately following cervical spinal manipulation, an emergent vascular etiology was suspected. Initial non-contrast head CT showed no hemorrhage or acute infarct. CT angiography (CTA) of the head and neck revealed complete occlusion of the proximal right vertebral artery at the C3 level.

The diagnosis of vertebral artery dissection (VAD) was favored over chronic atherosclerotic occlusion or thromboembolic etiology due to several imaging characteristics: the abrupt tapering or “flame-shaped” cutoff of the vessel, the presence of a string sign, and focal vessel narrowing suggestive of a mural hematoma. The absence of significant atherosclerotic disease elsewhere in the vertebrobasilar circulation and the acute symptomatic presentation following neck manipulation further supported dissection as the primary diagnosis.

The patient was managed conservatively with blood pressure control and supportive rehabilitation, ultimately returning to her baseline level of function with only mild residual aphasia. This favorable outcome underscores the importance of early recognition, appropriate imaging, and multidisciplinary management, including neurologic, vascular, and rehabilitative care, in optimizing recovery following VAD.

Given the continued debate surrounding the safety of cervical manipulation, clinicians should maintain a high index of suspicion for VAD in patients presenting with new neurologic symptoms after chiropractic care. Thorough history-taking, especially regarding recent neck trauma or manipulation, remains essential. Practitioners performing CSMT should also be aware of the potential risks, particularly in older patients or those with known vascular risk factors or congenital vascular anomalies.

## Conclusions

This case highlights a rare presentation of vertebral artery dissection (VAD) manifesting as isolated expressive aphasia immediately following cervical spinal manipulation therapy (CSMT), in the absence of neck pain or other typical posterior circulation symptoms. The patient’s favorable recovery through conservative medical management-despite complete occlusion of the vertebral artery-underscores the potential for non-interventional treatment in select, clinically stable patients with adequate collateral perfusion.

Additionally, the incidental discovery of a left middle cerebral artery (MCA) aneurysm prompted important multidisciplinary considerations for future surveillance, demonstrating how comprehensive vascular imaging can uncover clinically silent but actionable findings. The absence of formal screening before manipulation and the lack of identifiable risk factors in this patient further emphasize the need for cautious patient selection and heightened clinical vigilance when considering high-velocity cervical techniques.

Ultimately, this case reinforces the importance of prompt vascular imaging and a tailored, interdisciplinary approach to stroke care-particularly in atypical presentations invites further discussion on standardizing risk assessment before cervical manipulation.

## References

[1] Tavakoli SG, Britt TB, Agarwal S (2025). Vertebral artery dissection. StatPearls Publishing.

[2] Whedon JM, Petersen CL, Schoellkopf WJ, Haldeman S, MacKenzie TA, Lurie JD (2023). The association between cervical artery dissection and spinal manipulation among US adults. Eur Spine J.

[3] Pantbalekundri N, Gaidhane SA, Malali S, Nelakuditi M (2024). Spontaneous bilateral vertebral artery dissection as a rare cause of posterior circulation stroke in a young patient. Cureus.

[4] Kaiser JT, Reddy V, Launico MV (2025). Anatomy, head and neck: cervical vertebrae. StatPearls Publishing.

[5] Arnold M, Bousser M (2005). Clinical manifestations of vertebral artery dissection. Front Neurol Neurosci.

[6] Gottesman RF, Sharma P, Robinson KA, Arnan M, Tsui M, Saber-Tehrani A, Newman-Toker DE (2012). Imaging characteristics of symptomatic vertebral artery dissection: a systematic review. Neurologist.

[7] Albuquerque FC, Hu YC, Dashti SR (2011). Craniocervical arterial dissections as sequelae of chiropractic manipulation: patterns of injury and management. J Neurosurg.

[8] Mitra A, Azad HA, Prasad N (2021). Chiropractic associated vertebral artery dissection: an analysis of 34 patients amongst a cohort of 310. Clin Neurol Neurosurg.

